# Clinical and Epidemiological Features of Typhoid Fever in Pemba, Zanzibar: Assessment of the Performance of the WHO Case Definitions

**DOI:** 10.1371/journal.pone.0051823

**Published:** 2012-12-20

**Authors:** Kamala Thriemer, Benedikt B. Ley, Shaali S. Ame, Jaqueline L. Deen, Gi Deok Pak, Na Yoon Chang, Ramadhan Hashim, Wolfgang Hellmut Schmied, Clara Jana-Lui Busch, Shanette Nixon, Anne Morrissey, Mahesh K. Puri, R. Leon Ochiai, Thomas Wierzba, John D. Clemens, Mohammad Ali, Mohammad S. Jiddawi, Lorenz von Seidlein, Said M. Ali

**Affiliations:** 1 International Vaccine Institute, Seoul, Korea; 2 Institute of Tropical Medicine, Antwerp, Belgium; 3 University of Vienna, Biocenter, Vienna, Austria; 4 Public Health Laboratory (Pemba), Ivo de Carneri, Chake Chake, Tanzania; 5 Ministry of Health and Social Welfare, Zanzibar, Tanzania; 6 Menzies School of Health Research, Casuarina, Northern Territory, Australia; 7 Duke University, KCMC Collaboration, Moshi, Tanzania; Instituto de Higiene e Medicina Tropical, Portugal

## Abstract

**Background:**

The gold standard for diagnosis of typhoid fever is blood culture (BC). Because blood culture is often not available in impoverished settings it would be helpful to have alternative diagnostic approaches. We therefore investigated the usefulness of clinical signs, WHO case definition and Widal test for the diagnosis of typhoid fever.

**Methodology/Principal Findings:**

Participants with a body temperature ≥37.5°C or a history of fever were enrolled over 17 to 22 months in three hospitals on Pemba Island, Tanzania. Clinical signs and symptoms of participants upon presentation as well as blood and serum for BC and Widal testing were collected. Clinical signs and symptoms of typhoid fever cases were compared to other cases of invasive bacterial diseases and BC negative participants. The relationship of typhoid fever cases with rainfall, temperature, and religious festivals was explored. The performance of the WHO case definitions for suspected and probable typhoid fever and a local cut off titre for the Widal test was assessed. 79 of 2209 participants had invasive bacterial disease. 46 isolates were identified as typhoid fever. Apart from a longer duration of fever prior to admission clinical signs and symptoms were not significantly different among patients with typhoid fever than from other febrile patients. We did not detect any significant seasonal patterns nor correlation with rainfall or festivals. The sensitivity and specificity of the WHO case definition for suspected and probable typhoid fever were 82.6% and 41.3% and 36.3 and 99.7% respectively. Sensitivity and specificity of the Widal test was 47.8% and 99.4 both forfor O-agglutinin and H- agglutinin at a cut-off titre of 1∶80.

**Conclusions/Significance:**

Typhoid fever prevalence rates on Pemba are high and its clinical signs and symptoms are non-specific. The sensitivity of the Widal test is low and the WHO case definition performed better than the Widal test.

## Introduction

Diagnosing typhoid fever among the many causes of fever in sub-Saharan Africa is a huge challenge [1]. With declining malaria rates and more widespread deployment of *Haemophilus influenzae* and pneumococcal vaccines in many parts of the continent, the relative proportion of typhoid fever patients among patients with severe febrile illness presenting for care is likely to increase. The current gold standard for diagnosis of typhoid fever is blood culture. However blood culture requires a well- equipped laboratory, skilled staff, and may take up to seven days [2]. In the light of limited laboratory facilities in many developing countries, the diagnosis of typhoid fever remains a challenge. Specific clinical signs [3], [4], [5] or cheap and accurate point of care tests have remained elusive [2], [6], [7] and clinical algorithms are controversial because of their limited generalizability [8]. In addition to laboratory-confirmed typhoid fever the World Health Organization provides case definitions for suspected and probable typhoid fever for use during surveillance [9].

We conducted a prospective fever surveillance study in three district hospitals in Pemba Island, Zanzibar, Tanzania to determine potential clinical signs associated with typhoid fever, to assess the performance of WHO case definitions for suspected and probable typhoid fever and a local cut off titre for the Widal test, and, to assess potential fluctuation of *Salmonella* Typhi occurrence by seasonality.

## Methods

The study was implemented between March 2009 and December 2010 in all three district hospitals of Pemba Island, Zanzibar. Surveillance in Chake Chake district hospital was 22 months (March 2009–December 2010), Mkoani district hospital was 20 months (May 2009–December 2010) and Wete district hospital was 17 months (August 2009–December 2010).

The screening and inclusion process has been described in detail elsewhere [10]. In brief, patients 2 months of age or older attending one of the three hospitals were screened for eligibility. Outpatients with a recorded temperature of ≥37.5°C (tympanic thermometer) and inpatients with a reported history of fever were eligible to participate. If patients provided signed consent a standard physical exam was performed and a 1–5 ml or a 10–12 ml blood sample was collected from children and adults respectively. The sample was used for culture, Widal test, haemoglobin and glucose testing (Hemocue, Ängelholm, Sweden). Blood for culture was inoculated into the blood culture bottles at the hospital and then transfered together with the remaining sample to the Public Health Laboratory in Chake Chakeon a daily basis. Upon arrival blood culture bottles were inserted into an automated blood culture machine (BACTEC 9050, BD, USA). The remaining blood samples were centrifuged and the serum was used for immediate Widal testing. Positive blood cultures were processed further according to standard procedures [11], gram negative isolates suspicious for *S*.Typhi were confirmed by API20E (Biomerieux, France) and serology (poly O, Vi and O9 group D antiserum; BD, USA). Widal tube agglutination test was done according to package insert (Span Diagnostics, India) using a dilution series of 1∶40–1∶1280 for O and H antigens. All technicians performing the Widal tests were blinded to the culture results.

Clinical data were entered into a personal digital assistant. Laboratory data was recorded on paper forms and double entered into a custom made relational database [12]. For the purpose of these analyses the following definitions were applied: Bacteremia was defined as fever with isolation of human pathogenic bacteria. Contaminants were defined as non-pathogenic bacteria deriving from the skin during blood collection. This included *Bacillus* spp., *Corynebacterium* and coagulase-negative *Staphylococci*. Diarrhea was defined as loose or watery stools ≥3 times per 24 h.

Temperature and rainfall data was obtained from the Tanzania Meteorological Agency, Chake Chake Airport, Pemba.

Performance of the Widal test was calculated considering blood culture as the gold standard. The following definitions were applied: sensitivity was the likelihood that the test was positive if the participant had a blood culture confirmed *S.* Typhi infection, and specificity was the likelihood that the test was negative if the blood culture had excluded an acute episode of typhoid fever. The positive predictive value (PPV) and negative predictive value (NPV) described the likelihood that the test result would describe the presence or absence of disease in the participant correctly.

Confidence intervals were calculated using exact methods. Local cut off titres were established using Receiver Operating Characteristics (ROC) by plotting 1-specificity (x-axis) versus sensitivity (y-axis) at all tested cut off titres. The best cut off was considered the point with the highest Youden Index (sensitivity+specificity −1), representing the cut off value with the highest number of true positive and the lowest number of false positive results. The area under the curves for O-agglutinins (TO) and H agglutinins (TH) were compared to identify which antigen provides superior performance. Similar to the evaluation of the Widal test, we assessed the sensitivity, specificity, PPV and NPV of the WHO definitions of a suspected (fever for at least 3 days) and probable (fever for at least 3 days and positive serodiagnosis) typhoid fever case using blood culture as the gold standard.

Chi-square test, Fisher's exact -test and Mann-Whitney U test were used for comparisons between clinical variables and blood culture outcome as appropriate. Due to the multiplicity of comparisons a p value<0.01 was chosen as a threshold for significance in the univariate analyses. Variables with a p value<0.3 where included in a multivariate logistic regression model to determine predictors for typhoid fever. Correlations coefficient with rainfall and temperature were calculated using Spearman ranks test. All analyses were performed using STATA 12 (StataCorp 12, College Station, TX, USA) and Microsoft excel (Microsoft Corp. VA, USA).

Ethical approval was obtained from the Zanzibar Medical Research and Ethics Committee and the International Vaccine Institute - Institutional Review Boards, South Korea. Written informed consent was obtained from all study participants or their legal guardians.

## Results

**Figure 1:**
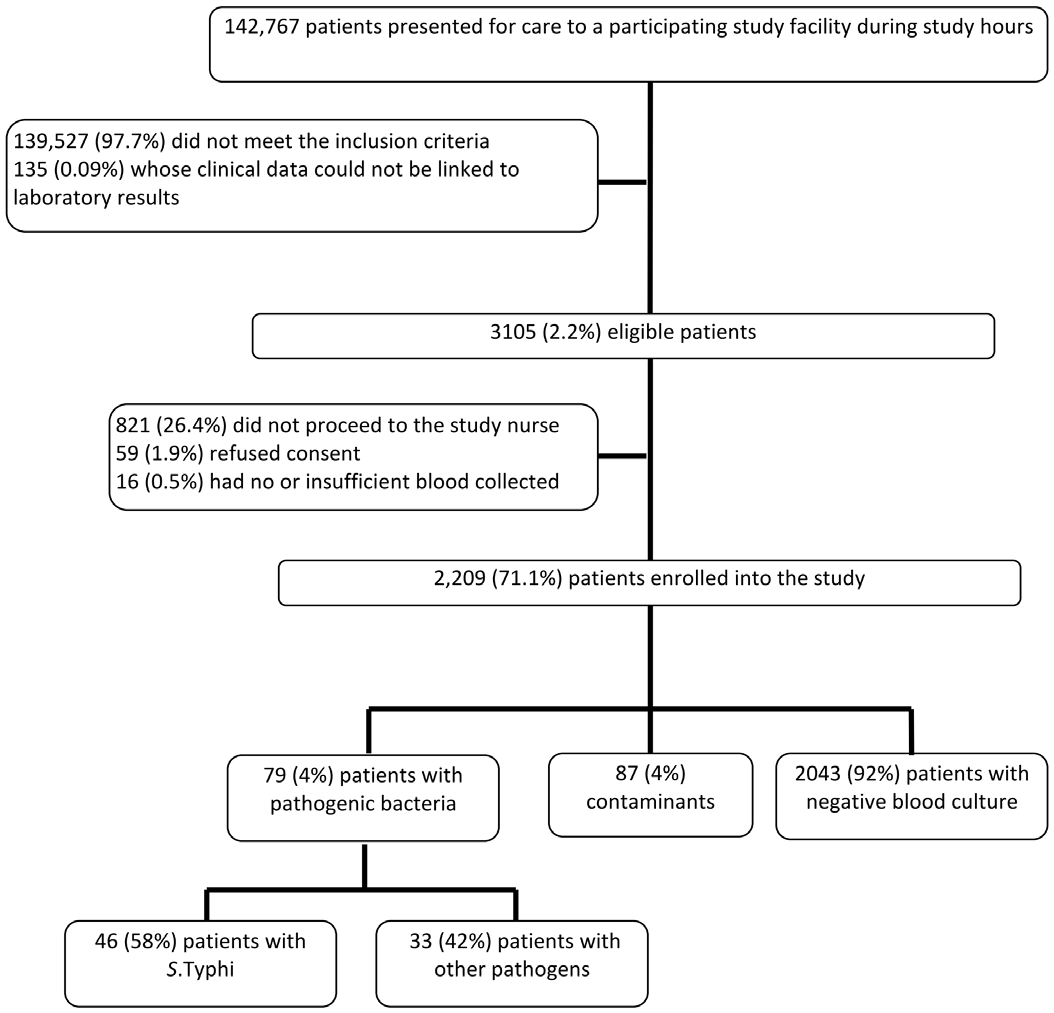
Consort chart of patients in the study.

We screened a total of 142,767 patients for eligibility and 2209 (1.5%) patients were enrolled into the study. From 79 (4%) patients were pathogenic bacteria isolated. *S.* Typhi was found to be the leading pathogen with 46 (58%) isolates, followed by *Streptococcus pneumoniae* (n = 12; 15%) (Figure 1).

Out of the 46 typhoid patients 28 (61%) were female. The median age of the typhoid patients was 16 years (range: 10 months – 58 years; mean: 17.7 years). Isolation rates were similar in all age groups. Two thirds (n = 30; 65%) of the typhoid fever patients were treated as outpatients and the remaining 16 typhoid fever cases were admitted. The admission rates were similar in the three hospitals (Table 1). Approximately one third of the patients with bacteremia were admitted as inpatients compared to one fifth of febrile patients who were not bacteremic (p = 0.017) (table 2). Inpatients were more likely to have *S.*Typhi isolated from their bloodstream than outpatients (3.5% versus 1.7%; p = 0.02).

**Table 1:** Proportion of In- and Outpatients with *S.*Typhi per hospital.

	Total number of *S.*Typhi cases	Number (%) of inpatients with *S.*Typhi	Number (%) of outpatients with *S.*Typhi	p*
**Chake Chake**	20	9 (45.0%)	11 (55.0%)	
**Mkoani**	21	5 (23.8%)	16 (76.2%)	0.37
**Wete**	5	2 (40.0%)	3 (60.0%)	
**Total**	46	16 (34.8%)	30 (65.2%)	

* using fisher's exact test.

**Table 2:** Proportion of In- and Outpatients with *S.*Typhi, other pathogens and no bacterial growth.

	Total	Inpatients	Outpatients	p*
***S.*** **Typhi (n = 46)**	46	16 (34.8%)	30 (65.2%)	
**Other pathogenic bacteria; (excl. contaminants) (n = 33)**	33	10 (30.3%)	23 (69.7%)	0.016
**No bacterial growth (incl. contaminants) (n = 2130)**	2130	422 (19.8%)	1708 (80.2%)	
**Total**	2209	433 (20.4%)	1689 (79.6%)	

* using chi^2^ test.

Direct comparison of clinical variables obtained on admission between patients diagnosed with typhoid fever and those with other bacterial infections or no bacterial growth in the blood culture showed significant differences only in the duration of fever before admission (5.3 days with a range of 0 to 14 days versus 4.0 days; p = 0.008). Although not significant a trend for lower haemoglobin levels in typhoid fever patients compared to other febrile cases was detected (10.5 g/dl versus 11.3 g/dl; p = 0.013)(Table 3). Differences in other variables including glucose levels, heart rate, and presence of diarrhea, convulsions, wheeze, crepitations and jaundice were not statistically significant. A logistic regression model for those variables revealed duration of fever as the only significant predictor for typhoid fever [OR of 1.07 (95% CI 1.01–1.13)].

**Table 3:** Clinical signs and symptoms of typhoid fever patients compared to other febrile patients.

	S.Typhi (n = 46)	Other bacteria (incl. cont.)+no bacterial growth (n = 2163)	P*
Mean age (n = 2209)	17.17	18.43	0.6297
Number (%) of male (n = 2209)	18 (39%)	1000 (46%)	0.339
Mean Glu (n = 1583)	115.67	108.19	0.0786
Mean Hb (n = 1938)	10.5	11.3	0.0130
Mean Temperature in C° (n = 2207)	37.75	37.80	0.5554
Mean duration of fever in days (n = 2021)	5.33	4.01	**0.0088**
Mean heart rate per minute (n = 2189)	85.89	89.39	0.2760
Mean weight in kg (n = 2205)	38.63	36.05	0.4581
N (%) history of fever (n = 2185)	44 (96%)	2074 (97%)	0.415
N (%) with cough (n = 2201)	18 (39%)	929 (43%)	0.59
N (%) with breathing difficulties (n = 2208)	1 (2%)	79 (4%)	1.0
N (%) with diarrhea (n = 2208)	9 (20%)	295 (14%)	0.249
N (%) with vomiting (n = 2194)	8 (17%)	289 (13%)	0.440
N (%) with convulsions (n = 2208)	1 (2%)	11 (0.5%)	0.224
N (%) with coma (n = 2207)	0 (0%)	2 (0.09%)	0.959
N (%) with pitting oedema (n = 2199)	0 (0%)	4 (0.18%)	0.919
N (%) with severe wasting (n = 2209)	0 (0%)	12 (0.55%)	0.776
N (%) skin pitch (n = 2197)	0 (0%)	6 (0.27%)	0.881
N (%) sunken eyes (n = 2188)	0 (0%)	1 (0.05%)	0.979
N (%) with signs of shock (n = 2154)	0 (0%)	2 (0.09%)	0.961
N (%) with lymphadenopathy (n = 2103)	0 (0%)	10 (0.48%)	0.813
N (%) with low chest indrawing (n = 2198)	0 (0%)	7 (0.32%)	0.862
N (%) with inspiratory stridor (n = 2193)	0 (0%)	9 (0.42%)	0.826
N (%)with wheeze (n = 2183)	2 (4.5%)	186 (8.7%)	0.228
N (%) with crepitations (n = 2177)	1 (2.2%)	108 (5%)	0.319
N (%) with jaundice (n = 2192)	0 (0%)	109 (5%)	0.093
N (%) with stiffness of the neck (n = 2179)	0 (0%)	5 (0.23%)	0.899

* using Chi^2^, Fisher exact and Mann-Whitney U test as appropriate; significance level <0.01.

When patients with negative blood cultures are excluded from the analyses duration of fever [OR = 1.27 (95%CI 1.09–1.47)] and weight [OR = 1.02 (95% CI 1.01–1.04)] were independently significant variables predicting the presence of typhoid fever.

**Figure 2:**
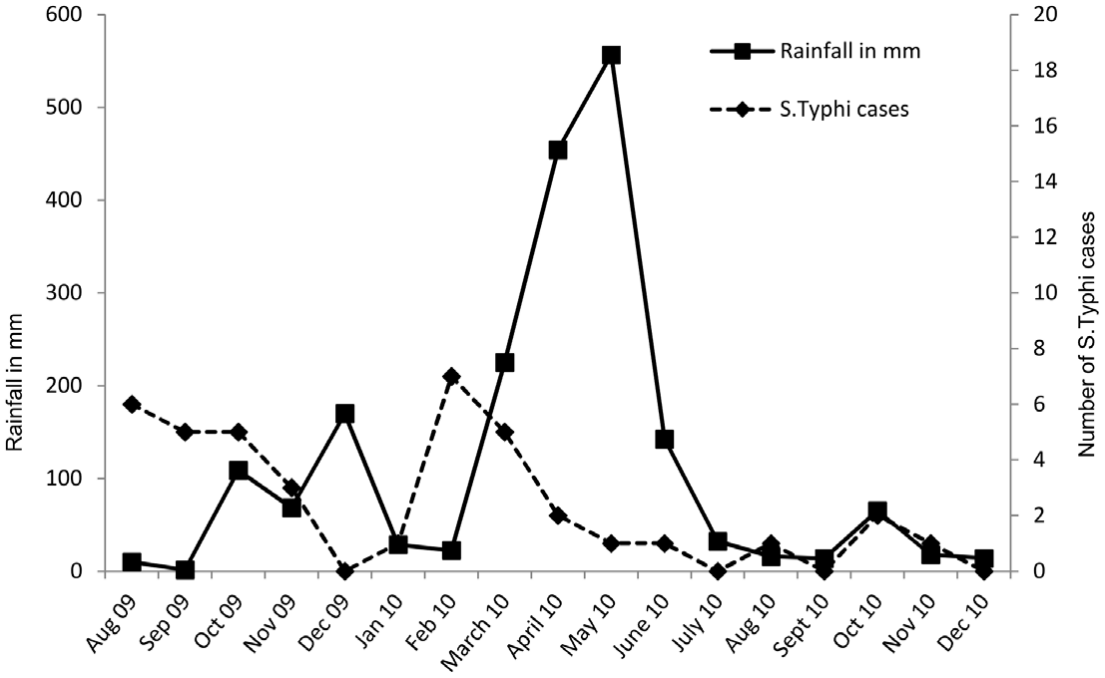
*S.*Typhi cases and rainfall in mm per month.

**Figure 3:**
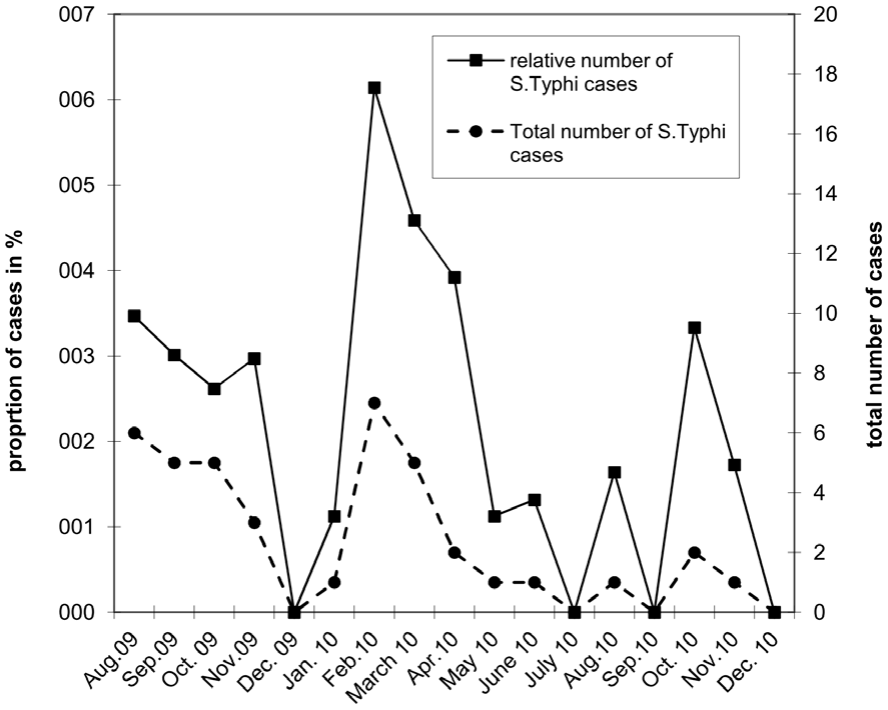
Total number of S.Typhi cases and proportion of S.Typhi cases in all fever cases per month.

A significant inverse correlation (p = 0.03) was observed between *S.* Typhi cases per month and average monthly rainfall for the first year of surveillance (March 2009 until March 2010, Chake Chake hospital only) but not for the entire study period (p>0.05) (Figure 2). No significant correlation was found with average monthly temperature or rainfall (p>0.05). The total number of *S.* Typhi cases as well as the proportion of typhoid fever cases among patients with bacteremia varied significantly over time (p = 0.046) with peaks in August 2009, February/March 2010 and October 2010 (Figure 3).

A total of 2179 (98.6%) samples were tested with the Widal tube agglutination test. The overall *S.* Typhi positivity rate was 2.1% (95CI: 1.5 to 2.8). When calculating best performance using Youden Index we found best indicators for O and H at a titre of 1∶80 with a sensitivity of 47.8% (95CI: 932.9 to 63.1) and 47.8% (95CI: 32.9 to 63.1) and a specificity of 99.4% (95CI: 98.6 to 99.7) and 99.4% (95CI: 99.0 to 99.7) for O and H respectively. The positive predictive value (PPV) and negative predictive value (NPV) were calculated to be 62.9% (95CI: 46.3 to 76.8) and 98.9% (95CI: 98.33 to 99.3) for O antigen and 64.7% (95CI: 46.6 to 80.3) and 98.9% (95CI: 98.3 to 99.3) for the H antigen (Table 4). The area under the ROC curve for O and H antigen were similar with 0.737.

**Table 4:** Sensitivity, Specificity, PPV and NPV of the Widal Test according to different cut off titres.

	Sensitivity	Specificity	PPV	NPV
TO, n = 2179	TP/(TP+FN)	TN/(TN+FP)	TP/(TP+FP)	TN/(TN+FN)
	95%CI*	95%CI*	95%CI*	95%CI*
	8.7	99.9	66.7	98.1
**1∶320**	4/46	2131/2133	4/6	2131/2173
	2.4–20.7	99.7→100.0	22.3–95.7	97.4–98.6
	30.4	99.9	82.4	98.5
**1∶160**	14/46	2130/2133	14/17	2130/2162
	17.7–45.8	99.6→100.0	56.6–96.2	97.9–99.0
	47.8	99.4	62.9	98.9
**1∶80**	22/46	2120/2133	22/35	2120/2144
	32.9–63.1	98.6–99.7	46.3–76.8	98.3–99.3

	Sensitivity	Specificity	PPV	NPV
TH, n = 2179	TP/(TP+FN)	TN/(TN+FP)	TP/(TP+FP)	TN/(TN+FN)
	95%CI*	95%CI*	95%CI*	95%CI*
	23.9	99.9	84.6	98.4
**1∶320**	11/46	2130/2132	11/13	2130/2165
	12.6–38.8	99.6→100.0	54.6–98.1	97.8–98.9
	39.1	99.9	85.7	98.7
**1∶160**	18/46	2129/2132	18/21	2129/2157
	25.1–54.6	99.5→100.0	63.7–97.0	98.1–99.1
	47.8	99.4	64.7	98.9
**1∶80**	22/46	2120/2132	22/34	2120/2144
	32.9–63.1	99.0–99.7	46.5–80.3	98.3–99.3

* exact method.

The WHO case definition of a suspected case of typhoid fever had a sensitivity of 72.1% and 82.6%, a specificity of 36.2% and 36.3% and PPV of 4.0% and 2.7% and NPV of 97.2% and 99.0% for predicting bacteraemia and typhoid fever, respectively (table 5). The WHO case definition of a probable case of typhoid fever had a sensitivity of 41.3%, a specificity of 99.7%, a PPV of 73.1% and an NPV of 98.7% for both TO and TH titre at a cut off level of 1∶80 for predicting typhoid fever. There was a significant difference between the accuracy of the WHO definition for suspected cases of typhoid fever and the definition of probable cases as well as the accuracy of the Widal test alone (p<0.05).

**Table 5:** Sensitivity, Specificity, PPV and NPV of the WHO case definitions of typhoid fever.

Suspected case of typhoid fever (≥3 days of fever) n = 2209				
Gold standard Isolation in blood culture of:	Sensitivity	Specificity	PPV	NPV
	TP/(TP+FN)	TN/(TN+FP)	TP/(TP+FP)	TN/(TN+FN)
	95%CI*	95%CI*	95%CI*	95%CI*
***S.*** **Typhi**	82.6%	36.3%	2.7%	99.0%
	38/46	785/2163	38/1416	785/793
	71.6–93.6	34.3–38.3	1.8–3.5	98.3–99.7
**All pathogenic bacteria**	72.1%	36.2%	4.0%	97.2%
	57/79	771/2130	57/1416	771/793
	62.3–82.0	34.2–38.2	3.0–5.1	96.1–98.4

Probable case of typhoid fever (≥3 days of fever and positive serology) n = 2179				
Gold standard Isolation in blood culture of *S.*Typhi and:	Sensitivity	Specificity	PPV	NPV
	TP/(TP+FN)	TN/(TN+FP)	TP/(TP+FP)	TN/(TN+FN)
	95%CI*	95%CI*	95%CI*	95%CI*
**Widal at O agglutination positive (cut off 1∶80)**	41.3%	99.7%	73.1%	98.7%
	19/46	2126/2133	19/26	2126/2153
	27.1–55.5	99.4–99.9	56.1–90.1	98.3–99.2
**Widal at H agglutination positive (cut off 1∶80)**	41.3%	99.7%	73.1%	98.7%
	19/46	2126/2133	19/26	2126/2153
	27.1–55.5	99.4–99.9	56.1–90.1	98.3–99.2

* wald method.

## Discussion

Typhoid fever was found to be non-specific in terms of clinical signs and symptoms compared to other febrile episodes and similar to Mtove *et al.* [5] we observed that patients with an acute episode of typhoid fever would attend hospital significantly later than patients with other bacterial infections and patients where no bacteria were isolated from the blood stream. Duration of fever was therefore the only significant predictor for typhoid fever.

The majority of typhoid fever cases were treated as outpatients, only 35% of typhoid fever cases were admitted to hospital. We found that participants suffering from typhoid fever experienced only mild fever with an average body temperature of 37.7°C on admission. This might be attributed to the nature of the study, allowing for early detection and treatment and is unlikely to reflect to course of the disease in the normal setting.

We assessed the seasonality of infections within the study setting, but did not detect any specific pattern. We observed increased numbers of cases aggregated in short time intervals, separated by periods of low or no *S.* Typhi infections. There was no significant correlation between rainfall, temperature, or religious festivals and number of cases throughout the study period.

In the absence of specific clinical signs and symptoms we assessed the performance of the widely used Widal tube agglutination test. Applying Youden Index we found a cut off titre of 1∶80 both for TO and TH provided the best performance for sensitivity and specificity in the study setting. However the clinical more relevant and prevalence dependent predictive values performed best at a cut off titre of 1∶160 or above for both antigens in this study setting.

A sensitivity of more than 80% for the WHO case definition of suspected typhoid cases was superior to the sensitivity of the Widal test and superior to the sensitivity of other rapid diagnostics tests such as Tubex [13] at the cost of poor specificity below 40%. When including Widal results according to the case definition of probable typhoid fever cases by the WHO, sensitivity dropped to little over 40%, while with these more stringent criteria specificity improved to nearly 100%. Comparing accuracy of the WHO definitions and the Widal test we found that neither the Widal test nor clinical signs and symptoms were sufficiently specific to diagnose typhoid fever reliably in Pemba. There is no major advantage to use WHO case definition of probably cases when the Widal test is already in use.

A major limitation, which may have contributed to these findings, is the relatively small sample of typhoid fever cases under investigation. It is possible that a larger sample of typhoid fever cases would detect statistically significant relationships. Secondly, since the sensitivity of blood cultures for S. Typhi is rather poor, especially in children were the collected blood volume was relatively low an unknown fraction of the culture negative patients were likely to be bacteremic. These misclassifications could further mask differences between patient groups.

## Conclusion

The clinical features of typhoid fever on Pemba are non-specific. The widely used Widal test performed rather poorly.The WHO case definition for suspected cases performed better than the Widal test, however with a high rate of false positive results. Neither rainfall, seasonality nor big religious festivals appeared to be risk factors associated with infection. Blood culture remains the gold standard for typhoid fever diagnosis, but its usage is limited by the lack of equipment, funds and experience of laboratory technicians in many developing countries. An affordable, accurate, and reliable diagnostic test for typhoid fever would be of great benefit in many resource poor countries and preventive efforts such as vaccination should be considered especially in settings with high disease burden.
