# Correction: Clinical and Epidemiological Features of Typhoid Fever in Pemba, Zanzibar: Assessment of the Performance of the WHO Case Definitions

**DOI:** 10.1371/annotation/18ab2cda-9bb4-45ca-ae9c-517654f8160d

**Published:** 2013-06-07

**Authors:** Kamala Thriemer, Benedikt Ley, Shaali S. Ame, Jaqueline L. Deen, Gi Deok Pak, Na Yoon Chang, Ramadhan Hashim, Wolfgang Hellmut Schmied, Clara Jana-Lui Busch, Shanette Nixon, Anne Morrissey, Mahesh K. Puri, R. Leon Ochiai, Thomas Wierzba, John D. Clemens, Mohammad Ali, Mohammad S. Jiddawi, Lorenz von Seidlein, Said M. Ali

The second author's name was spelled incorrectly. The correct name is: Benedikt Ley. The correct citation is: Thriemer K, Ley B, Ame SS, Deen JL, Pak GD, et al. (2012) Clinical and Epidemiological Features of Typhoid Fever in Pemba, Zanzibar: Assessment of the Performance of the WHO Case Definitions. PLoS ONE 7(12): e51823. doi:10.1371/journal.pone.0051823 

